# Controlled release of celecoxib inhibits inflammation, bone cysts and osteophyte formation in a preclinical model of osteoarthritis

**DOI:** 10.1080/10717544.2018.1482971

**Published:** 2018-06-12

**Authors:** A. R. Tellegen, I. Rudnik-Jansen, B. Pouran, H. M. de Visser, H. H. Weinans, R. E. Thomas, M. J. L. Kik, G. C. M. Grinwis, J. C. Thies, N. Woike, G. Mihov, P. J. Emans, B. P. Meij, L. B. Creemers, M. A. Tryfonidou

**Affiliations:** a Department of Clinical Sciences of Companion Animals, Faculty of Veterinary Medicine, Utrecht University, Utrecht, The Netherlands;; b Department of Orthopaedics, University Medical Centre Utrecht, Utrecht, The Netherlands;; c Department of Rheumatology and Clinical Immunology, University Medical Centre Utrecht, Utrecht, The Netherlands;; d Department of Pathobiology, Faculty of Veterinary Medicine, Utrecht University, Utrecht, The Netherlands;; e DSM Biomedical, Geleen, the Netherlands;; f Department of Orthopaedics, University Medical Centre Maastricht, Maastricht, The Netherlands

**Keywords:** Drug delivery, polyesteramide microspheres, synovitis, sclerosis, cartilage, COX-2

## Abstract

Major hallmarks of osteoarthritis (OA) are cartilage degeneration, inflammation and osteophyte formation. COX-2 inhibitors counteract inflammation-related pain, but their prolonged oral use entails the risk for side effects. Local and prolonged administration in biocompatible and degradable drug delivery biomaterials could offer an efficient and safe treatment for the long-term management of OA symptoms. Therefore, we evaluated the disease-modifying effects and the optimal dose of polyesteramide microspheres delivering the COX-2 inhibitor celecoxib in a rat OA model. Four weeks after OA induction by anterior cruciate ligament transection and partial medial meniscectomy, 8-week-old female rats (*n* = 6/group) were injected intra-articular with celecoxib-loaded microspheres at three dosages (0.03, 0.23 or 0.39 mg). Unloaded microspheres served as control. During the 16-week follow-up, static weight bearing and plasma celecoxib concentrations were monitored. Post-mortem, micro-computed tomography and knee joint histology determined progression of synovitis, osteophyte formation, subchondral bone changes, and cartilage integrity. Systemic celecoxib levels were below the detection limit 6 days upon delivery. Systemic and local adverse effects were absent. Local delivery of celecoxib reduced the formation of osteophytes, subchondral sclerosis, bone cysts and calcified loose bodies, and reduced synovial inflammation, while cartilage histology was unaffected. Even though the effects on pain could not be evualated directly in the current model, our results suggest the application of celecoxib-loaded microspheres holds promise as novel, safe and effective treatment for inflammation and pain in OA.

## Introduction

Osteoarthritis (OA) is the most common form of arthritis in humans. It is estimated that 18% of women and 10% of men over the age of 60 years suffer from OA (Cross et al., 2014). With aging of the population and the increasing prevalence of obesity, the incidence of OA is rising concurrently (Holt et al., 2011). OA can result in joint pain, stiffness and functional limitations, negatively influencing quality of life (Lane et al., 2011).

Pain in OA is related to several associated disease processes, of which cartilage degeneration, synovial inflammation and peri-articular bone reaction, including bone cysts and osteophyte formation play an important role (Loeser et al., 2012; Glyn-Jones et al., 2015). Inflammation of the synovial lining results in the production of pro-inflammatory mediators and degradative enzymes, thereby mediating pain and facilitating further joint degeneration. These pro-inflammatory mediators are associated with the progression of OA pain (Baker et al., 2010) and disease (Roemer et al., 2011). Moreover, subchondral bone changes are considered increasingly important in OA. Bone marrow lesions (BML) and subchondral bone cysts appear early in the disease process (Alliston et al., 2017) and are visible as regions of hyperintense marrow signal in fluid-sensitive MRI image sequences (Kon et al., 2016). Both have been associated with joint pain (Yusuf et al., 2011) and disease progression (Taljanovic et al., 2008; Tanamas et al., 2010). Other peri-articular bone changes include subchondral bone sclerosis and the formation of osteophytes (Loeser et al., 2012). Osteophytes may impair joint mobility and can cause pain by impinging surrounding structures (Sofat et al., 2011). As such, OA is considered a disease of the whole joint and successful therapeutic strategies should involve disease-modifying drugs that exert effects at multiple levels (Karsdal et al., 2008).

Oral nonsteroidal anti-inflammatory drugs (NSAIDs) are frequently used to inhibit pain and inflammation in OA (Wolfe et al., 2000; Moskowitz et al., 2007). It has been suggested that celecoxib, a selective COX-2 inhibitor and the first drug to be approved for oral administration in OA, has disease-modifying properties. Cyclo-oxygenase-2 (COX-2) expression is upregulated in OA joints, resulting in pro-inflammatory mediators, such as prostaglandin E2 (PGE_2_) (Pincus 2001). The latter is associated with inflammation of the synovium, cartilage degeneration and the sensitization to pain (Wang et al., 2013). Clinical studies have already proven that celecoxib is effective in relieving OA pain (Pincus et al., 2004; McCormack 2011). *In vitro* studies even suggested a protective effect of celecoxib on OA cartilage (Mastbergen et al., 2002, de Boer et al., 2009). In line with this, cartilage of patients orally treated with celecoxib contained significantly more proteoglycans compared to cartilage of patients treated with the nonspecific COX inhibitor indomethacin (de Boer et al., 2009). Moreover, celecoxib was able to prevent synovial hyperplasia and bone destruction both *in vitro* and *in vivo* (Zweers et al., 2011). A reduction in osteophytes was found when rats with surgically induced OA were treated with celecoxib orally (Panahifar et al., 2014).

Although COX-2 inhibition is effective in attenuating the symptoms of OA, longitudinal clinical studies associated oral COX-2 inhibitors at a higher oral dose with increased cardiovascular risk (Solomon et al., 2017). To overcome these issues, local biomaterial-based delivery of celecoxib can be a suitable treatment alternative, by providing prolonged drug exposure (Larsen et al., 2008). Local drug delivery not only prevents systemic side effects, it also ensures optimal exposure in the joint cavity and avoids drug binding to systemic molecules and drug modifications that can limit efficacy when the drug is administered systemically (Evans et al., 2014).

To facilitate local delivery and sustained local exposure to drugs, biomaterial carriers can be used. Although a few *in vivo* studies have investigated intra-articular (IA) delivery platforms of celecoxib in both healthy (Petit et al., 2015) and OA joints (Dong et al., 2013), the optimal dose range of celecoxib remains unknown. Polyesteramide (PEA) microspheres are very suitable for local sustained drug delivery, given their favorable mechanical and thermal properties and extended drug release profiles (Andres-Guerrero et al., 2015; Rudnik-Jansen et al., 2017). The local safety of IA injection of celecoxib-loaded PEA microspheres (PEAMs) has previously been confirmed in healthy and OA rat knee joints 12 weeks after injection (Janssen et al., 2016). There, degradation of PEAMs was shown to be faster in OA compared to healthy joints, suggesting celecoxib-loaded PEAMs as a potent drug delivery system with autoregulatory behavior (Janssen et al., 2016). However, no therapeutic effects were noted, which may have been due to the dosage used. Therefore, the aim of this study was to assess the safety and efficacy of PEA microspheres loaded with a wide dose range of celecoxib *in vivo* in osteoarthritic rat joints, starting with twice the dosage used in the aforementioned study. By increasing the loading dose of celecoxib, we expect more pronounced tissue modulating effects on OA progression.

## Material and methods

### Synthesis of the polyesteramide copolymer

The biomaterial in this study is a biodegradable poly(ester amide) (PEA) based on α-amino acids, aliphatic dicarboxylic acids and aliphatic α-ω diols. The selected PEA comprises three types of building blocks randomly distributed along the polymer chain. The polymer was synthesized according to a procedure reported previously (Katsarava et al., 1999). Brieﬂy, the polymer was prepared via solution polycondensation of di-p-toluenesulfonic acid salts of bis-(α-amino acid) α,ω- diol diesters, lysine benzyl ester and di-N-hydroxysuccinimide sebacate in anhydrous DMSO. The use of pre-activated acid in the reaction allows polymerization at low temperature (65 °C) affording side-product free polycondensates and predictable degradation products. The polymer was isolated from the reaction mixture in two precipitation steps.

### Polymer characterization

^1^H NMR spectra were obtained on a Bruker Avance 500 MHz Ultrashield NMR; samples were recorded in DMSO d_6_. Molecular weight and molecular weight distributions of PEA were determined by GPC equipped with RI detector. Samples were dissolved in THF at a concentration of approximately 5 mg/mL and were run at a ﬂow rate of 1 mL/min at 50 °C. The molecular weights were calibrated to a narrow polystyrene standard calibration curve, using Waters Empower software.

### Synthesis and characterization of (celecoxib-loaded) microspheres

Polyesteramide polymer was dissolved in dichloromethane. To generate celecoxib (CXB) loaded PEA microspheres (PEAMs), the drug was added. After homogenization, the solution was sonicated in a water bath for 3 minutes. The PEA-CXB solution was then emulsified in 20 ml of water phase (PVA 1 wt%, NaCl 2.5 wt%) by the use of an ultraturrax, stirring at 8000 rpm for 3 minutes. After emulsification, particles were hardened overnight under air flow. Before washing, particles were cooled with an ice-bath for 1 hour and washed with Tween 80. Excess of surfactant was removed by centrifugation. Before freeze-drying to remove residual solvent, particles were suspended in Tween 80 in order to reach the right concentration of particles per volume. 15 and 70 mg/ml are aimed for CXB loaded- and unloaded PEAMs. Once dried, the particles were weighted in individual HPLC vials to the approximate amount of 15 or 70 mg respectively and γ-sterilized on dry ice.

**Figure 1.**
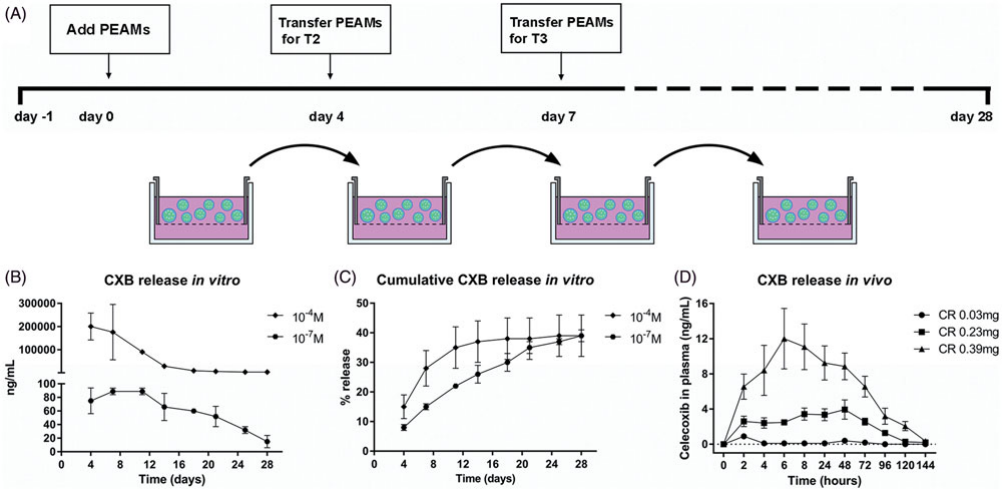
(A) Setup of *in vitro* release of celecoxib (CXB). Absolute (B) and cumulative (C) celecoxib release from PEA microspheres (PEAMs) in plain culture medium: after 28 days, 40% was released. *N* = 2 per time point. (D) CXB release in plasma after intra-articular injection in osteoarthritic rat knee joints with three different loadings as indicated. *N* = 6 per group. Plasma CXB concentrations were significantly different (*p* < .001) between groups at all timepoints, except for T = 144h. Data depicted as average ± SD.

Particle characteristics are described in Table 2. To measure celecoxib release in vitro, celecoxib-loaded PEAMs were dispersed in culture medium (hgDMEM + Glutamax, 1966; Invitrogen) and placed in Transwell® baskets (pore size 0.4 µm, polycarbonate membrane, 3413; Corning Life Sciences). Two concentrations, of celecoxib-loaded PEAMs were utilized: 10^−7 ^M and 10^−4 ^M corresponding to 1.33 µg CXB/mL and 1.33 mg CXB/mL. Medium was changed twice a week, aliquots (700 μL) were obtained at day 4, 7, 11, 14, 18, 21, 25 and 28 and stored at −20 °C (Figure 1(A)). For celecoxib measurements, medium samples were lyophilized for 3 hours and dissolved in 50 µL buffer (180719; Neogen Corporation) overnight at 4 °C prior to analysis. Celecoxib release from the PEAMs was measured by ELISA (180719; Neogen Corporation) following manufacturer’s instructions.

## Animal study

### Study setup

This study was approved by the Dutch ethics committee for laboratory animal use (protocol #2014.III.10.086). Female, 8-week-old Sprague Dawley rats (Charles River laboratories, The Netherlands) were allowed to acclimatize for 7 days and housed in groups (3 to 4 rats) in polycarbonate cages with wire tops, wood chip bedding, and access to *ad libitum* food and tap water.

OA was induced unilaterally through anterior cruciate ligament transection (ACLT) and partial medial meniscectomy (pMMx) in the left knee of 24 rats (Gerwin et al., 2010). Details on group size calculations are described in Supplementary file 1. Completeness of ACLT was confirmed intra-operatively by a positive drawer sign. Pain management included 4 mg/kg carprofen and 0.03 mg/kg buprenorphine subcutaneously prior to surgery, buprenorphine was continued b.i.d. for three days. Animals were monitored daily for signs of discomfort and were weighed weekly. Four weeks later, rats were randomly divided into four groups with six rats per group (Table 1). On two consecutive days (day 0 and day 1), rats received IA injection with unloaded PEAMs (OA control) or PEAMs loaded with 0.015 mg/25 µL (low dose; LD), 0.115 mg/25 µL (medium dose; MD) or 0.195 mg/25 µL (high dose, HD) celecoxib. On day 0, 100 μL blood was collected five times every 2 hours (starting from directly prior to injection) to monitor systemic release of celecoxib. On day 1–7, blood was collected once daily (starting from 24 hours after injection) and thereafter once weekly until termination of the study. After sixteen weeks, rats were euthanized with CO_2_.

**Table 1.** Overview of experimental groups and microsphere loading.

Treatment	No of rats	Total dosage (mg/kg)	Particle concentration [mg/ml]	Loading drug [wt%]	Total drug injected [mg]	Mean particle size [μm]	Span
Unloaded	6	N/A	70	N/A	N/A	33.4	1.47
LD-CXB	6	0.13	15	4.1%	0.03	36.6	1.47
MD-CXB	6	0.99	70	6.5%	0.23	35.7	1.62
HD-CXB	6	1.67	70	11.2%	0.39	36.9	1.51

LD: low dose; MD: middle dose; HD: high dose; CXB: celecoxib; N/A: not applicable.

**Table 2.** Polymer characterization.

	Mn (kDa)	Polydispersity index (PDI)	Glass transition temperature (Tg)	Relative monomer ratio A:B:C
PEA III Ac Bz	70	1.70	54 °C	0.30:0.27:0.43

The relative ratio between the polymer building blocks was determined by 1H NMR. Tg of the polymer was determined under dry conditions.

### Longitudinal measurements in vivo

EDTA-plasma was collected in capillary blood collection tubes (T-MQK Capiject, Terumo Medical Corporation) and stored at −80 °C until further use. Celecoxib was measured in plasma samples diluted 1:5 with buffer by ELISA (Neogen). A calibration curve ranging from 0.4 to 100 ng/mL celecoxib (C-1502, LC Laboratories) was measured in spiked EDTA-plasma of healthy rats from unrelated experiments.

Hind limb weight distribution as an index of pain was obtained with an incapacitance tester (Linton Instrumentation) before and 3 weeks after OA induction, and weekly after IA injections, as described previously (van Buul et al., 2014). The average of 5 measurements was used to calculate the weight on the affected limb as a percentage of total weight distributed by both hind limbs (Figure 3(A)).

### Postmortem analysis

Directly postmortem, micro-computed tomography (µ-CT) scans were acquired with a Quantum FX µ-CT Imaging System (Perkin Elmer) to assess subchondral bone. Scans with an isotropic voxel size of 15 µm, at a voltage of 90 kV, a current of 180 μA and a field of view of 42 mm were created. Subchondral plate thickness, volume and porosity were measured in the cortical bone of the medial and lateral tibial plateau in coronal scans as previously described by de Visser et al. (2017). With the use of ImageJ software, the regions of interest (ROI) were selected. Bone was segmented from the µ-CT datasets with a local threshold algorithm (Bernsen, radius 5) from the coronal sections. Regions of interests were manually drawn for a total of 90 slides, starting in the caudal side of the knee joint from the point where the medial and lateral compartments of the tibial epiphysis unite, onwards to the cranial side of the knee joint. ROIs were manually drawn in the subchondral bone of the lateral and medial compartments of the tibia plateau, resulting in data on mean subchondral plate thickness (mm) and subchondral bone volume fraction (BV/TV) representing the ratio of trabecular bone volume (BV, in mm^3^) to endocortical tissue volume (TV, in mm^3^), the mean trabecular bone thickness (mm) and trabecular bone volume fraction (BV/TV). The average distance (mm) between individual trabeculae (‘spacing’) was also recorded. In addition to subchondral bone changes, all knee joints were also assessed for the presence of subchondral bone sclerosis, osteophytes, subchondral bone cysts (SBCs) and loose bodies according to the method of Panahifar (Panahifar et al., 2014). Briefly, SBCs were defined as round structures with no trabeculae, recognizable from black structures on µ-CT. Their presence was scored in the sagittal plane in the tibia, primarily on coronal plane, with grade 0 indicating absence of SBCs and grade I presence of SBCs. Loose bodies in the synovial capsule based on their number where 0 = none, 1 = 1 loose body, 2 = 2 loose bodies and 3 = 3 or more loose bodies. Subchondral sclerosis was evaluated at both medial and lateral sides in the femur and tibia in the sagittal plane based on a three scale score (a maximum score of six for each bone). Sclerosis was defined as a solid mineralized region with no distinct trabecular structure. The depth of sclerosis was measured on sagittal CT sections, from the articular surface along the diaphysis and the maximum value was reported. Baseline data were analyzed and depth of up to 0.3 mm was considered normal thickness of subchondral bone plate. Osteophytes were scored separately for femur, tibia and patella at four regions. The maximum depth of osteophyte perpendicular to bone was measured and scored in a two scale score (maximum of eight for each bone). Depth of less than 0.2 mm was considered ambiguous and scored 0. The reference plane for scoring femur and tibia was axial and for the patella, coronal. The diameter of SBCs was also measured in ImageJ (version 1.50e). All treated knees per treatment group were used for analyses by a blinded observer, together with six randomly selected non-treated control knees, leading to six knees per group. A blinded observer (AT) analyzed all µ-CT scans of the knees together.

Co-morbidities were explored with the aid of several parameters, including body weight reflecting well-being of the experimental animal during the study and postmortem examination of the rats. Internal organs were assessed macroscopically and histologically to rule out systemic effects or co-morbidities, by blinded observers (RT, MK). Histopathologic analysis was performed according to standard protocols and tissue samples were fixed in 4% buffered formalin, embedded in paraffin and cut at 4 µm before routine staining with hematoxylin and eosin (H&E). Bacteriological testing was performed when there was macroscopic and cytological evidence of bacterial infection. Both necropsy and histological assessment were performed by operators blinded to the treatment administered. All organs were reviewed by RT and MK and the degree of hepatic vacuolization was subsequently scored using the grading scheme proposed by Hardisty and Eustis (1990). Briefly, grades 1 (mild) to 5 (severe) based on the number of fields affected out of the fourteen randomly chosen high-powered fields that were viewed. Cytoplasmic vacuolization was identified as being lacey (glycogen) or micro- or macro-vacuolar (fat).

All hind limbs were dissected and fixed in 4% v/v formalin for 7 days at RT. The histological preparations and analysis were performed according to the OARSI guidelines and scored on coronal 5 μm thick sections of EDTA-decalcified knee joints at 100 μm intervals (Gerwin et al., 2010). Cartilage quality was assessed focusing on the following OARSI components: cartilage matrix loss width, cartilage degeneration, cartilage degeneration width, osteophytes, synovial inflammation and calcified cartilage, subchondral bone damage and growth plate thickness. To detect macrophages, CD68 immunohistochemistry (IHC) was performed (Supplementary file 2). Collagen X IHC was performed to visualize hypertrophic differentiation of chondrocytes. The growth plate served as an internal positive control. The expression patterns of inducible nitric oxide synthase (i-NOS) and folate receptor beta (FR-β) were used to distinguish between M1 and M2 macrophages. Photographs were obtained of cartilage and synovial tissue adjacent to the medial tibia plateau. In ImageJ, the total surface (pixels) of the synovial tissue or cartilage on digital photographs was obtained by manually selecting the region of interest. Positive DAB staining was quantified in that ROI. Expression was quantified by the % of positive surface.

### Statistical analysis

Statistical analysis was conducted using IBM SPSS statistics 24.0 and R studio (*RStudio* 3.3.1). Normality of the data was checked by assessing Q-Q plots, histograms and Shapiro–Wilk tests. The effect of treatment on weight distribution was analyzed using the Wilcoxon’s signed rank test. One-way ANOVA and Kruskall–Wallis tests were used to analyze µ-CT data and histological scores. Differences in plasma celecoxib concentration were analyzed by a Cox proportional hazard model, considering ‘donor’ as random effect and ‘time’ and ‘treatment’ as fixed effects. *p* < .05 was considered significant after correcting for multiple testing with Benjamini&Hochberg False Discovery Rate *post hoc* tests. Effect sizes (ES) were retrieved as *Hedge’s g* for parametric data*:* medium; 0.5–0.8, large; 0.8–1.2 very large and >2 huge (Sawilowsky 2009). Differences were considered as relevant when *p* < .05 and/or ES was medium or larger when *p* value was <0.1. For nonparametric data, Cliff’s delta was assessed: 0.28 < ES <0.43, medium; 0.43 ≤ ES <0.7, large; ES ≥0.7, extra large (Vargha & Delaney, 2000). *p*-values and effect sizes for all comparisons made in this study are provided in Supplementary file 3.

## Results

### Polymer characterization

**Figure 2.**
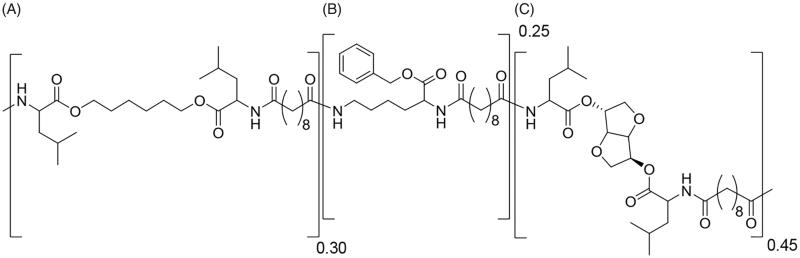
Structure of PEA III Ac Bz, random copolymer consisting of building blocks A, B and C.

The relative ratio between the polymer building blocks was determined by 1 H NMR. Tg of the polymer was determined under dry conditions. The average molecular weight of the polymer was 70 kDa, the polydispersity index (PDI) 1.70, the glass transition temperature (Tg) 54 °C and the relative monomer ratio of A:B:C was 0.30:0.27:0.43. The polymer used for this study is depicted in Figure 2.

### *In vitro* and *in vivo* release of celecoxib

*In vitro*, CXB-PEAMs demonstrated a sustained drug release (Figure 1(B)) with cumulative release of 40% after 28 days (Figure 1(C)). *In vivo*, the small volume of synovial fluid in rat knees did not allow for sampling to monitor local celecoxib release from PEAMs. Therefore, systemic plasma celecoxib concentrations were determined. Celecoxib was, dose-dependently, detectable until 120 hours after IA injection (Figure 1(D)).

### Hind limb weight distributions seemed to restore as a result of treatment with LD-celecoxib-PEAMs

**Figure 3.**
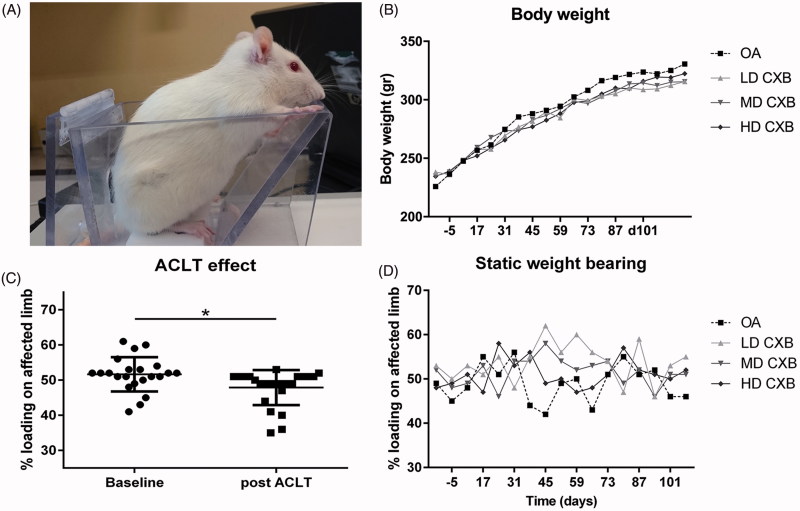
(A) Setup of the pressure plate measurements. (B) Body weight increased gradually in all groups during the course of the study. (C) Load bearing significantly decreased (*p* = .013) in operated joints 3 weeks after anterior cruciate ligament transection and partial medial meniscectomy (ACLT + pMMx). (D) Static weight bearing improved 6 weeks after OA induction, but only the LD-CXB-PEAMs could significantly enhanced weight bearing compared to OA control joints (*p* = .044). ACLT: Anterior cruciate ligament transection; OA: osteoarthritis (unloaded PEAM control group); LD CXB: low dose celecoxib; MD CXB: middle dose CXB; HD CXB: high dose CXB.

During the study period, average body weight increased gradually from 233 g (range 196-290 g) to 321 g (274–405 g), as expected. No differences were observed in body weight between treatment groups (Figure 3(B)). No treatment-related systemic abnormalities were found on necropsy confirming systemic safety. Before OA induction, rats bore weight on both hind limbs equally: 50.8%±6.7% (mean ± SD) (Figure 3(C)). Three weeks after ACLT + pMMx, weight distribution on the operated leg was significantly lower than the pre-operative situation (Figure 3(C), *p* = .013). One week after IA injection of (un)loaded-PEAMs and throughout the whole study period, only borderline significant differences compared to pretreatment values were found (*p* = .058). Post hoc tests revealed a significant increase in weight bearing of the affected leg only with LD-CXB-PEAMs (*p* = .044) vs. unloaded-PEAMs (Figure 3(D)).

### *Ex vivo* μ-CT revealed protective effects of prolonged celecoxib exposure on OA progression at the subchondral bone level

**Figure 4.**
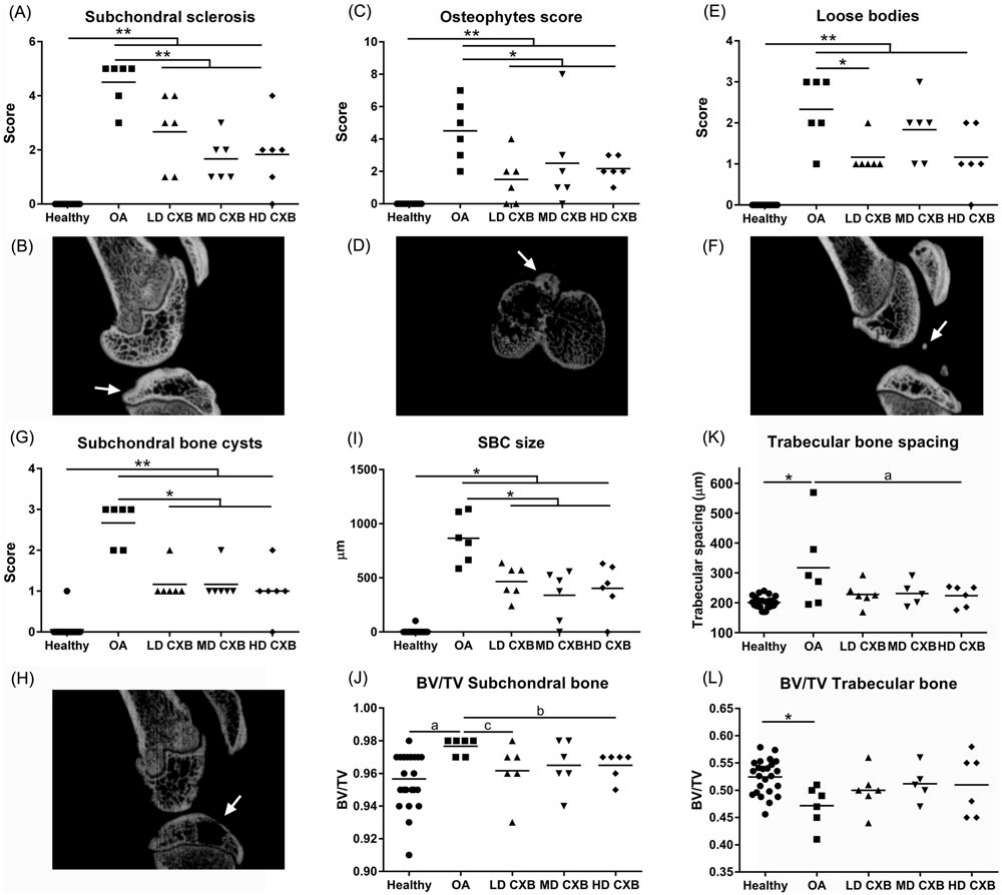
*Ex vivo* micro computed tomography (µ-CT) analysis of the medial tibia plateau. The induction of osteoarthritis (OA) led to an increase in subchondral sclerosis (A,B; *p* < .001), which was inhibited by the controlled release (CR) of celecoxib (CXB) (A; *p* = .007, *p* = .063, *p* = .003 for low, middle and high dose CXB (LD-, MD- and HD-CXB)). CXB-PEAMs exerted a protective effect on the formation of osteophytes (C,D; *p* = .006, *p* = .036 and *p* = .019 for LD-, MD- and HD-CXB) and the presence of loose bodies (E,F; *p* = .011, *p* = .24, *p* = .142) in OA joints. CR of celecoxib reduced the number of subchondral bone cysts (G,H; LD-CXB *p* = .002; MD-CXB *p* = .037; HD-CXB *p* = .002) in OA joints, and also resulted in smaller cysts (I; *p* = .0015, *p* = .002, *p* = .0018). The induction of OA resulted in increased bone volume of the subchondral bone plate (J; *p* = .059, ES 0.7), which was decreased by HD-CXB-PEAMs (*p* = .09, ES 0.8). Bone volume of the trabecular bone was decreased after OA induction (K, *p* = .027), but was not influenced by CXB-PEAMs. HD-CXB-PEAMs tended to prevent increase of trabecular bone spacing (K; *p* = .063, ES 0.65). *p < .05, **p < .01, a: medium effect size (ES); b: large ES; c: very large ES.

*Ex vivo* µ-CT was used to quantitatively evaluate the subchondral bone of the medial tibial plateau. All measured μ-CT parameters were significantly different in OA vs. healthy contralateral knees (Figure 4(A–L)), confirming the typical hallmarks of OA at subchondral bone level, that is increase in subchondral sclerosis (Figure 4(A,B); *p* < .001), osteophyte formation (Figure 4(C,D); *p* < .001), the presence of calcified loose bodies (Figure 4(E,F); *p* < .001) SBCs (Figure 4(G,H); *p* = .001), whereas healthy knees had none. Moreover, lower porosity of the subchondral bone plate (Figure 4(J), *p* = .059, medium ES) and a decrease in bone volume of the trabecular bone (Figure 4(L); *p* = .027) beneath the subchondral plate, with a concurrent increase in trabecular spacing (Figure 4(K), *p* = .003) were detectable in OA joints.

Less subchondral sclerosis was demonstrated in OA knees treated with LD-, MD- and HD-CXB-PEAMs (Figure 4(A)), vs. unloaded-PEAMs (*p* < .001). Knee joints that received celecoxib-PEAMs contained significantly less osteophytes (Figure 4(C), *p* < .05 for LD-, MD- and HD-CXB-PEAMs) vs. unloaded-PEAMs. In OA knees treated with unloaded-PEAMs, significantly more SBCs were scored compared to knees treated with CXB-PEAMs (Figure 4(G), *p* < .05 for all dosages). Furthermore, SBC size was significantly smaller in OA knees treated with celecoxib-loaded-PEAMs vs. unloaded-PEAMs (Figure 4(I); *p* < .05 for all dosages). In OA knees, loose bodies were present (Figure 4(E,F)); LD-CXB-PEAMs lowered their numbers vs. unloaded PEAMs (Figure 4(E), *p* = .011). Furthermore, HD-CXB-PEAMs inhibited increase in subchondral BV/TV (Figure 4(J), *p* = .09, large ES), and tended to counteract an increase in trabecular bone spacing (Figure 4(L), *p* = .063, medium ES). No significant differences between treatment groups were detected in trabecular thickness of subchondral nor trabecular bone. Growth plate thickness was unaffected by OA induction and treatment with celecoxib (data not shown).

### Histologic evaluation of cartilage integrity and synovial inflammation showed anti-inflammatory effects of celecoxib-releasing microspheres but no inhibition of cartilage degeneration

**Figure 5.**
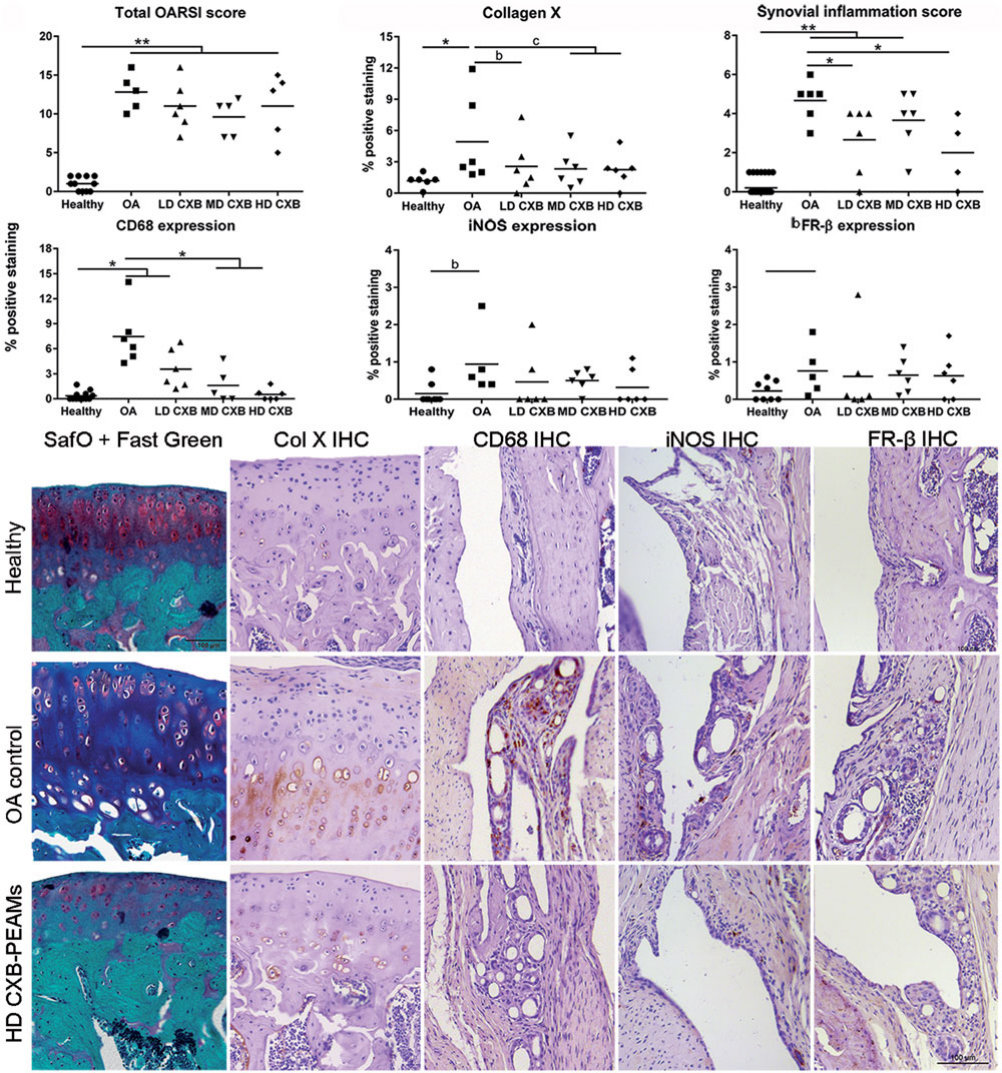
Histological grading and immunohistochemical analysis of the medial tibial. OA induction led to an increase in OARSI score, collagen X deposition and synovial inflammation. Celecoxib-loaded PEAMs were able to decrease synovial inflammation, macrophage presence and collagen X deposition. *p < .05; **p < .01; a: medium effect size (ES); b: large ES; c: very large ES. OA: osteoarthritic control knees; LD: low dose; MD: middle dose; HD: high dose celecoxib. HD CXB-PEAMs: high dose celecoxib-polyesteramide microspheres; IHC: immunohistochemistry.

Histomorphometrical measurements were performed in the most affected region, that is, the medial tibia plateau. The OARSI score (Figure 5) was significantly higher in OA versus healthy contralateral knees (*p* < .01). Treatment with celecoxib-loaded PEAMs had no effect within the OA joints. The synovitis score was significantly increased in OA knees treated with unloaded-PEAMs vs. healthy (*p* < .001) and HD-CXB-PEAMs (*p* = .028). CD68 immunopositive cells, a general macrophage marker, were rarely seen in healthy knees. In OA control joints treated with unloaded-PEAMs, significantly more CD68 immunopositivity was found in the synovial perivascular regions vs. healthy controls (*p* < .001). There was a dose-dependent decrease in CD68 immunopositivity with increasing celecoxib loading dose as indicated by the significantly lower CD68 immunopositivity in MD-CXB (*p* = .016) and HD-CXB-PEAMs (*p* = .005) vs. unloaded-PEAMs. Given these distinct differences, the presence of M1/M2 macrophages was further profiled by evaluating iNOS and FR-β immunopositivity in consecutive sections. The expression of M1 related iNOS was upregulated in OA versus healthy contralateral joints (*p* = .012) and there was a substantive significant decrease in iNOS expression in the HD-CXB-PEAMs (*p* = .068, large ES). No significant differences in M2-related FR-β expression was noted between treatment groups, although FR-β expression seemed increased in OA vs healthy contralateral joints (*p* = .072, large ES).

Collagen X immunopositivity in the tibial cartilage of OA knees injected with unloaded-PEAMs was increased (*p* = .047) vs. healthy contralateral joints (*p* = .04), indicating hypertrophic differentiation. Controlled release of celecoxib seemed to inhibit collagen X deposition (*p* < .1; very large ES for LD-, MD- and huge ES for HD-CXB-PEAMs).

## Discussion

In the present *in vivo* study, the effects of sustained release of celecoxib from PEAMs, administered intra-articularly in a rat OA model were evaluated. Local or systemic adverse effects were absent in the (celecoxib-)PEAM-injected joints, indicating that the platform is safe to apply over a wide range of celecoxib loading. Systemic exposure of celecoxib after intra-articular injection was only detectable in the first 5 days after IA delivery, with substantially lower circulatory values than after oral administration (Ma et al., 2015). In addition, local treatment with celecoxib-loaded PEAMs inhibited the OA bone phenotype as demonstrated by a decrease in subchondral bone sclerosis, osteophytes, calcified loose body formation, and SBCs. Even more so, celecoxib-loaded PEAMs reduced local joint inflammation. Considering the demonstrated role of synovial inflammation and peri-articular bone changes in OA-related pain, local sustained delivery of celecoxib is a disease modifying drug that could alleviate pain.

Prolonged local exposure to celecoxib inhibited subchondral bone changes and osteophyte formation, characteristic for OA. An increasing body of evidence points to an interplay between bone and cartilage in OA (Karsdal et al., 2008), even suggesting a key role for subchondral bone in OA development (Botter et al., 2011). Targeting bone changes may be an effective strategy to reduce symptoms and disease progression in OA, as bone marrow lesions, bone cysts, osteophytes and bone shape were associated with structural progression and pain in patients with knee OA (Tanamas et al., 2010; Barr et al., 2015). The mechanism by which CXB inhibits OA bone changes may at least partially be related to its inhibitory effect on hypertrophic differentiation (Welting et al., 2011). The process of OA at least partially recapitulates chondrocyte hypertrophic differentiation during endochondral ossification (Dreier 2010), which was also observed in the present OA model where significantly more collagen X in the degenerating cartilage was found. Notably, local delivery of CXB-PEAMs partially counteracted cartilage collagen type X deposition, while it was even more effective in slowing progression of OA-related subchondral bone changes and osteophyte formation. This is in accordance with previous reports exploring the disease-modifying effects of oral celecoxib administration (Panahifar et al., 2014).

In parallel to beneficial effects at the subchondral level, local delivery of celecoxib reduced the number and size of SBCs. SBCs are cavitary lesions that contain fibrous tissue and fluid, but can ossify in later stages (Kon et al., 2016). They arise at locations where cartilage damage is most severe and are associated with activated osteoclasts and osteoblasts (Li et al., 2013). Notably, human patients suffering from OA with associated BMLs/SBCs appear to have more pain and an increased risk for joint replacement surgery (Barr et al., 2015; Kon et al., 2016). The inhibition of cyst formation by local sustained delivery of celecoxib suggests a second route toward inhibition of OA-associated pain in addition to the inhibition of synovial inflammation. As celecoxib exerts direct effects on bone by inhibiting NF-κB-dependent osteoclastogenesis and osteoclast activation and indirect effects by reducing RANKL production by OA chondrocytes (Zweers et al., 2011), this mechanism may account for the decreased number and size of SBCs in the present study.

This study also provided evidence for the inhibitory effect of local and sustained drug celecoxib delivery on osteophyte formation. From a clinical perspective, osteophytes can cause a decrease in joint range of motion and can also be a source of pain, by vascularization and associated innervation, and by impinging adjacent structures. Pain due to impingement can be an indication for surgical intervention for several joints such as the hip, shoulder and ankle joint, and it is recommended to remove osteophytes during arthroplasty surgery. Short-term outcome for cheilectomies is favorable, but osteophytes tend to recur in the long-term (Wong et al., 2016). In this respect, local prolonged exposure to celecoxib could slow down osteophyte formation or prevent recurrence after surgical removal.

Corroborating to the observed effects at the subchondral bone and peri-articular level, a single injection of CXB-PEAMs seemed to harness the synovial inflammatory process on the long term as indicated by the improvement of the synovitis score and reduction of infiltrating inflammatory macrophages 16 weeks after injection. In line with these findings, a previous study employing the same rat OA model and CXB-PEAMs demonstrated reduction of total PGE_2_ levels in knee homogenates, although improvement of synovitis at the histological level was not detected (Janssen et al., 2016). The latter could be attributed to the fact that half the loading dose of celecoxib per knee joint was used, compared to the lowest concentration employed in the current study. Indeed, celecoxib can inhibit synovial inflammation and proteolytic enzyme production through inhibition of the COX-2 and the NF-κB pathway *in vivo* (Zweers et al., 2011). In addition, celecoxib also inhibits proliferation of synovial fibroblasts, reducing synovial hyperplasia and potentially slowing down synovitis-mediated OA progression (Zweers et al., 2011).

Although beneficial effects of celecoxib on subchondral bone, osteophytes and synovium were found, protective effects on cartilage histology were absent. We cannot exclude that the absence of a chondroprotective effect could be attributed to the specific OA rat model used. In contrast to the monosodium iodoacetate (MIA) model, the ACLT model does not give rise to significant differences in gait parameters and pain-related behavior, while quick irreversible degenerative changes at the tissue level do occur (Ferland et al., 2011; Maerz et al., 2016). Moreover, lately evidence has been accumulating that inflammation may play a minor role in OA-associated cartilage degeneration. In a collagenase-induced OA model, IL-1α and IL-1β were not involved in cartilage destruction (van Dalen et al., 2017) and in clinical trials, where inhibition of TNF-α or IL-1β, key players in inflammation, had disappointing effects (Philp et al., 2017). In line with this, inflammation in the present rat OA model is low-grade, indicating that the role of inflammatory mediators in cartilage degeneration could be of minor importance.

Altogether, the present study showed that by fine-tuning the loading dose of the PEAM-based drug delivery platform, the disease modifying effects of celecoxib were further improved. Clear beneficial effects were exerted on the bone phenotype of OA. It remains to be investigated whether the controlled and local release of celecoxib also effectively inhibits pain-related inflammation. However, the inhibition of synovial inflammation and the inhibition of bone cysts and osteophyte formation suggests local delivery of celecoxib may be an effective treatment for both disease-modifying as well as OA-associated pain.

## Supplementary Material

Supplemental Material
