# Supplementary material for: Controlled release of celecoxib inhibits inflammation, bone cysts and osteophyte formation in a preclinical model of osteoarthritis
Source: Drug Deliv. 2018 Jun 12;25(1):1438–47. doi: 10.1080/10717544.2018.1482971 (PMC6058666; doi:10.1080/10717544.2018.1482971)
Supplement: Supplemental Material [file IDRD_A_1482971_SM5564.docx]

**Supplementary file 1. Power analysis and group size calculation for the *in vivo* experiment.**

To determine the total number of animals used in the *in vivo* experiment, and the number of animals in each experimental group, a power analysis was performed in G*Power 3.1.9.2. An a priori analysis was performed using an ANOVA, to compute the required sample size, given an effect size of 1, and an α of 0.008. An α of 0.008 was used as we had to correct for multiple comparisons: 6 comparisons in total results in an α of 0.05/6 = 0.008. A total of 24 animals was calculated (6 animals per experimental group), with a power of 0.85.

**Supplementary file 2. Details of the primary antibodies and the immunohistochemistry protocols employed.**

Deparaffinization was established through xylene (2x5 min) and graded ethanol (96, 80, 70, 60%, 5 min each), followed by two rinses of TBS+0.1% Tween (TBST0.1%, 2x5 min). Antigen retrieval was performed, followed by endogenous peroxidase inhibition for 5 min and pre-incubation with blocking buffer for 30 min at RT. Thereafter, sections were incubated with primary antibody at 4°C overnight (table 1). The EnVision-HRP detection system (Dako) was applied for 30 min at RT followed by incubation with streptavidin conjugated with horseradish peroxidase for 30 min at RT. All antibodies were visualized with the liquid DAB+ substrate chromogen (Dako). With the aid of ImageJ, immunopositivity was calculated as the % of positive staining in the total synovial area. Respective isotype controls confirmed specificity for the staining.

**Table 2.** Details on immunohistochemistry protocols.

| **Name** | **Manufacturer** | **Origin** | **Antibody Ig fraction** | **Antigen retrieval** | **Block** | **Dilution 1^st^ antibody** | **Secondary antibody** |
| --- | --- | --- | --- | --- | --- | --- | --- |
| CD68 | Abcam, ab31630 | Human recombinant | Mouse Mab | None | 1:10 goat serum / PBST | 0.2 μg/mL | EnVision K4001, Dako |
| Collagen X | Quartett, 2031501005 | Human recombinant | Mouse Mab IgG1 | 0.1% Pepsin, 20 min @37°C and HAse 10mg/mL, 30 min @37°C | 1:10 goat serum / PBST |  | EnVision K4001, Dako |
| Folate receptor β |  | Human recombinant | Rabbit Pab IgG | 0.01M citrate buffer pH 6.0 30 min @70°C | PBS-BSA 5% | 1:500 | X0903, Dako |
| NOS2 (iNOS) | Santa Cruz Bio-technology, SC-7271 | Human recombinant | Mouse Mab IgG1 | 0.1% Pepsin, 20 min @37°C | 0.3% H_2_O_2_; PBS-BSA 5% | 1:1000 | EnVision K4001, Dako |

Mab: monoclonal antibody; Pab: polyclonal antibody; HAse: bovine hyaluronidase 4 450 IU/mg, adjusted to pH 5 with 0.1M HCl.; PBST: 6 Phosphate buffered saline 0.1% Tween-20.

**Supplementary file 3.** Effect sizes (ES), ES’s confident intervals (99.9%, between brackets) and P values for the different analyses performed in the study with P-values between 0.05 and 0.1.

| **Experiment** | **Comparison** | **Effect size** | **P value** | **Type** |
| --- | --- | --- | --- | --- |
| Trabecular thickness subchondral bone | OA vs healthy | 0.21 |  | Hedge’s G |
|  | OA vs LD-PEAMs | 0.04 |  |  |
|  | OA vs MD-PEAMs | 0.15 |  |  |
|  | OA vs HD-PEAMs | 0.87 |  |  |
| Trabecular thickness trabecular bone | OA vs healthy | 0.27 |  | Hedge’s G |
|  | OA vs LD-PEAMs | 0.49 |  |  |
|  | OA vs MD-PEAMs | 0.18 |  |  |
|  | OA vs HD-PEAMs | 0.30 |  |  |
| BV/TV subchondral bone | OA vs healthy | 0.68 (0.85-2.20) | 0.059 # | Hedge’s G |
|  | OA vs LD-PEAMs | 1.27 (0.36-2.90) | 0.08 # |  |
|  | OA vs MD-PEAMs | 0.48 (-1.99-1.03) | 0.09 # |  |
|  | OA vs HD-PEAMs | 0.85 (0.70-2.41) | 0.08 # |  |
| BV/TV trabecular bone | OA vs healthy | 0.37 (-1.13-1.87) | 0.027 * | Hedge’s G |
|  | OA vs LD-PEAMs | 0 (-1.49-1.49) | 0.86 |  |
|  | OA vs MD-PEAMs | 0.19 (-1.30-1.68) | 0.79 |  |
|  | OA vs HD-PEAMs | 0.16 (-1.33-1.65) | 0.78 |  |
| Trabecular bone spacing | OA vs healthy | 0.82 (0.73-2.37) | 0.003 ** | Hedge’s G |
|  | OA vs LD-PEAMs | 0.50 (-2.01-1.01) | 0.093 # |  |
|  | OA vs MD-PEAMs | 0.47 (-1.98-1.04) | 0.128 |  |
|  | OA vs HD-PEAMs | 0.65 (0.95-2.08) | 0.071 # |  |
| Collagen X IHC | OA vs healthy | 1.26 (0.37-2.88) | 0.047 * | Hedge’s G |
|  | OA vs LD-PEAMs | 0.67 (-2.20-0.86) | 0.380 |  |
|  | OA vs MD-PEAMs | 0.82 (0.73-2.73) | 0.064 # |  |
|  | OA vs HD-PEAMs | 0.87 (0.69-2.43) | 0.052 # |  |
| iNOS IHC | OA vs healthy | 0.60 | 0.012 * | Cliff’s delta |
|  | OA vs LD-PEAMs | 0.40 | 0.177 |  |
|  | OA vs MD-PEAMs | 0.34 | 0.126 |  |
|  | OA vs HD-PEAMs | 0.34 | 0.068 # |  |
| FR-β IHC | OA vs healthy | 1.01 (0.57-2.14) | 0.072 # | Hedge’s G |
|  | OA vs LD-PEAMs | 0.22 (-1.71-1.27) | 0.63 |  |
|  | OA vs MD-PEAMs | 0.28 (-1.77-1.22) | 0.81 |  |
|  | OA vs HD-PEAMs | 0.31 (-1.80-1.19) | 0.56 |  |

Color labels for Hedge’s g: *none* (ES≤0.01), no fill; *very small* (0.01≤ES<0.2), purple; *small* (0.2≤ES<0.5), light blue; *medium* (0.5≤ES<0.8), yellow; *large* (0.8≤ES<1.2), green; *very large* (1.2≤ES<2), orange; and *huge* (ES≥2), red. Color labels for Cliff’s delta: *small* (ES<0.28), light blue; *medium* (0.28≤ES<0.43), yellow; *large* (0.43≤ES<0.7), green; and very l*arge* (ES≥0.7), red. (# p<0.1; * p<0.05; ** p<0.01).
